# Use of Autologous Platelets for Lamellar Macular Hole Repair

**DOI:** 10.1155/2019/1471754

**Published:** 2019-05-19

**Authors:** Andres Gonzalez, Sarina Amin, Omar Iqbal, Stephen Myles Potter, Syed Gibran Khurshid

**Affiliations:** ^1^University of Florida College of Medicine, Department of Ophthalmology, Gainesville, FL, USA; ^2^University of Florida College of Medicine, Gainesville, FL, USA

## Abstract

The consensus of lamellar macular hole repair generally entails vitrectomy with internal limiting membrane with/without epiretinal membrane peeling with gas tamponade, although the risk of a full thickness macular hole remains. In this case report, we investigate the role of the regenerative properties of autologous platelets in the repair of a lamellar macular hole with pars plana vitrectomy, an autologous platelet plug, and 12% C3F8 without prone posturing. All three patients in this case report experienced visual improvement along with anatomic closure of the lamellar macular hole. Further randomized studies with larger sample sizes will contribute to the existing data regarding this procedure and its outcomes.

## 1. Introduction

A lamellar macular hole (LMH) is a term originally suggested by Gass in 1975 to encompass a macular lesion resulting from cystoid macular edema [1]. Today, a LMH refers to partial-thickness defects of the macula that present on optical coherence tomography (OCT) with an irregular foveal contour and with separation of the inner from the outer layers in the fovea [2]. The prognosis and visual acuity of patients with a LMH is usually good, and most cases have historically been managed conservatively. Vision can deteriorate in a certain subset of patients, in which intervention may be indicated [2]. The most frequently employed surgical standard technique for LMH repair is a pars plana vitrectomy (PPV) with internal limiting membrane (ILM) with/without epiretinal membrane (ERM) peeling with/without endotamponade and prone posturing [2–7]. Excellent anatomical and functional outcomes have been obtained with this surgical procedure. Although uncommon, the risk of a full thickness macular hole (FTHM) remains; the highest reported incidence of a FTHM in studies of at least 20 patients was three patients (27.7%) [8]. Prone posturing can also be difficult in some patients with previous neck or back injury, vertebral fusion surgery, large body habitus, or respiratory issues. Given the real risk of a FTMH, the call for alternative techniques has been advocated for [9].

Platelets play an active role in tissue repair and glial cell proliferation given its growth factor milieu [10]. PPV with autologous platelets has been reported to achieve high anatomic and visual success rates in full-thickness macular hole repair [11–13], although there are no reports of this surgical technique being applied to lamellar macular holes. The purpose of this study was to assess the outcomes of lamellar macular hole repair with PPV, an autologous platelet plug, and 12% C3F8 without prone posturing.

## 2. Case Report

Three patients presented to the University of Florida Eye Clinic between April 2015 and March 2016 with a central scotoma from a LMH found on OCT. All patients were pseudophakic, and there were no other significant eye diseases present. Patients 1 and 2 likely developed a LMH from a history of cystoid macular edema after cataract surgery. The etiology for LMH formation in Patient 3 was unclear, although the patient was noted to have a posterior vitreous detachment on exam. No obvious operculum was seen in Patient 3 on exam. The OCT criteria defined by Witkin et al. were used as the reference for diagnosis of a LMH: (1) irregular foveal contour; (2) break in the inner fovea; (3) intraretinal split; and (4) intact foveal photoreceptors [14]. Using the 6-line radial pattern OCT, the LMH was differentiated from a macular pseudohole, a pseudocyst, and a full-thickness macular hole.

In all cases, a three-port 23-gauge PPV was performed by a single surgeon. Complete air-fluid exchange was performed. A 0.1mL autologous platelet plug was placed directly over the posterior pole at the macula. The air was then replaced with 12% octafluoropropane (C_3_F_8_). After surgery, patients were advised to remain in a supine position for one hour to allow the platelets to plug the LMH. No patients underwent ERM/ILM peeling. Patients were placed on a topical antibiotic-steroid drop for one month postoperatively. Follow-up examination occurred at one day, 1 month, 3 months, and 6 months.

**Figure 1:**
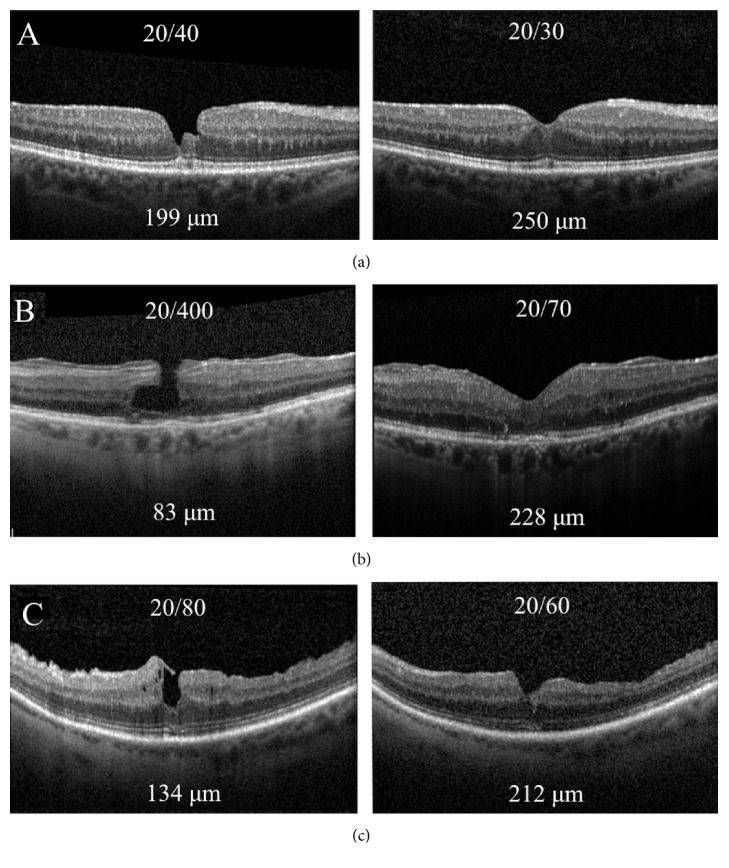
OCT images of Patients 1-3 (a-c) prior to surgery with respective visual acuities and CMT (left) and OCT images at last follow-up with respective visual acuities and CMT (right).

The average patient age at the time of surgery was 72 years (range, 70–74 years). There were two female patients and one male patient. Postoperative OCT analysis revealed that all patients had LMH closure (Figure 1) with repopulation of foveal tissue and reformation of foveal architecture. All patients were followed up for at least 6 months. There were no recurrences of the LMH during this time. No surgical complications were observed.

Mean logMAR VA improved from 0.733 (Snellen equivalent 20/108; see [20/40 to 20/400]) preoperatively to 0.399 (Snellen equivalent 20/50; see [20/30 to 20/70]) at 6 months. All patients had functional visual improvement of their central scotoma and all patients had anatomic closure of the LMH on OCT. Central macular thickness improved in all patients, from an average of 138.7 *μ*m preoperatively (range 83–199 microns) to 230 microns at 6 months (range 212–250 microns). See Table 1.

## 3. Discussion

Although excellent anatomic and functional results have been achieved with PPV with ILM with/without ERM peel with/without endotamponade [2–7], and the need for alternative surgical repairs has been advocated for given the uncommon but serious risk of a FTMH [9]. We present a case report of three patients that had a PPV, autologous platelet plug, and 12% C3F8 without prone posturing for LMH repair. Vitrectomy with autologous platelets has shown great success with macular holes [11–13]. There are no reports in the literature assessing this technique in LMH repair. Etiology of LMH was likely a history of cystoid macular edema after cataract surgery in Patient 1 and Patient 2 and possible hyaloidal traction from a posterior vitreous detachment in Patient 3. This case report showed an anatomical success rate of 100% along with visual improvement on Snellen and with respect to their central scotoma. No complications were noted after at least six months of follow-up for all patients. No patients received ERM/ILM peeling.

Müller cells are the principal glial cells of the retina and are integral for maintaining retinal architecture [15]. It is proposed that surgical decapitation of Müller cells' basement membrane stimulates glial cell proliferation and closure of a macular hole, hence the role of ILM peeling [10]. As an alternative, platelet suspensions have shown to achieve high anatomic success rates and good visual results for macular hole repairs [11–13]. The reason for this phenomenon is likely explained by the various growth factors present in platelets that have shown to stimulate Müller cells; these include platelet-derived growth factor (PDGF), epidermal growth factor (EGF), and fibroblast growth factor (FGF), among others [10]. Platelets become activated at the site of tissue injury, such as retinal tissue injury in a LMH. Activation releases these factors that support tissue repair by regulation of cell migration, mitosis, and differentiation [10]. We believe this is the pathophysiology as to why there was evidence of retinal glial cell proliferation, cellular repopulation of macular tissue, and reformation of normal foveal architecture in this case report. We believe that the regenerative properties of platelets reduce the risk of a FTMH. The disadvantage to using fresh autologous platelets is that intensive sterile preparation is needed.

We report another alternative treatment for LMH that involves PPV, autologous platelets, and endotamponade without postoperative prone posturing with good success in this pilot study. Further randomized studies with larger sample sizes will contribute to the existing data regarding this procedure and its outcomes.

## Figures and Tables

**Table 1:** Patient characteristics.

Patient	1	2	3
Age (Gender)	70 (Female)	74 (Male)	72 (Female)

Lens status	Pseudophakic	Pseudophakic	Pseudophakic

Preoperative CMT (*µ*m)	199	83	134

1-month CMT (*µ*m)	230		

3-month CMT (*µ*m)			

6-month CMT (*µ*m)	250	228	212

Pre-treatment Snellen VA (logMAR)	20/40 (sc) 0.30	20/400 (sc) (NI PH) 1.3	20/80 (sc) 0.60

1-month Snellen VA (logMAR)	20/60 (sc) 0.48	20/200 (sc) (NI PH) 1	20/100 (sc) 0.70

3-month Snellen VA (logMAR)		20/50 (BCVA) 0.40	20/100 (sc) 0.70

6-month Snellen VA (logMAR)	20/30 (sc) 0.18	20/70 (BCVA) 0.54	20/60 (sc) 0.48

Observation Period (months)	6	12	6

*∗* cc: with correction; *∗* sc: without correction.
